# Global Biobank Engine: enabling genotype-phenotype browsing for biobank summary statistics

**DOI:** 10.1093/bioinformatics/bty999

**Published:** 2018-12-05

**Authors:** Gregory McInnes, Yosuke Tanigawa, Chris DeBoever, Adam Lavertu, Julia Eve Olivieri, Matthew Aguirre, Manuel A Rivas

**Affiliations:** 1 Biomedical Informatics Training Program, Stanford University, CA, USA; 2 Department of Biomedical Data Science, Stanford University, CA, USA; 3 Institute for Computational and Mathematical Engineering, Stanford University, CA, USA

## Abstract

**Summary:**

Large biobanks linking phenotype to genotype have led to an explosion of genetic association studies across a wide range of phenotypes. Sharing the knowledge generated by these resources with the scientific community remains a challenge due to patient privacy and the vast amount of data. Here, we present Global Biobank Engine (GBE), a web-based tool that enables exploration of the relationship between genotype and phenotype in biobank cohorts, such as the UK Biobank. GBE supports browsing for results from genome-wide association studies, phenome-wide association studies, gene-based tests and genetic correlation between phenotypes. We envision GBE as a platform that facilitates the dissemination of summary statistics from biobanks to the scientific and clinical communities.

**Availability and implementation:**

GBE currently hosts data from the UK Biobank and can be found freely available at *biobankengine.stanford.edu.*

## 1 Introduction

Population-scale biobanks linking rich phenotype and molecular data are transforming the landscape of biomedical research. UK Biobank, a long-term prospective cohort study, has collected array-genotyped variants from 500 000 individuals and linked it with medical records, activity monitors, imaging and survey data (Sudlow *et al.*, 2015). Availability of these data enables researchers to perform analyses across a broad range of phenotypes at an unprecedented scale (Bycroft, 2017).

The value of large sequencing and genotyping efforts lies not only in primary publications but also in the dissemination of summary statistic data to the scientific community. Other large-scale efforts to sequence and analyze genetic data, such as ExAC and gnomAD (Lek *et al.*, 2016), have made data available to the scientific community at large available via web browsers (Karczewski *et al.*, 2017). Browsers serve as an effective communication tool that enable researchers around the world to interrogate genetic statistics of interest. Often, these tools limit the information shared to summary statistics which confers a decreased privacy risk for individuals included in the study (Erlich and Narayanan, 2014) and limits the computational resources required to interrogate the data. However, to date no such tool exists that offers researchers the opportunity to study the relationship between genotype and phenotype.

Here, we present Global Biobank Engine (GBE), a web-based tool that presents summary statistics resulting from analysis of genotype-phenotype associations derived from data in population-scale biobanks. GBE serves as a means to communicate scientific discoveries to the scientific community without requiring sharing of individual-level data. In particular, we present results from genome-wide association studies (GWAS) and phenome-wide association studies (PheWAS) for White British individuals (*n* = 337 199) in UK Biobank, gene-level phenotype associations, genetic correlations and others. Results for each analysis are pre-computed allowing for rapid browsing. Phenotypes currently available in the browser are those made available by UK Biobank, including cancer, disease status, family history of disease, medication, quantitative measures, as well as computational grouping of phenotypes based on self-reported data and ICD 10 codes from hospital in-patient record data (as described in DeBoever *et al.*, 2018a).

We encourage use of GBE but note that case-control results are provided as general guides and may not have been subjected to the data quality, statistical and population genetics review that would normally be required for publication of clinical inference.

## 2 Features

GBE serves as a platform to host summary statistics that explore different facets of biobank data. Here, we describe the features available.

### 2.1 Phenotype page

**Fig. 1.**
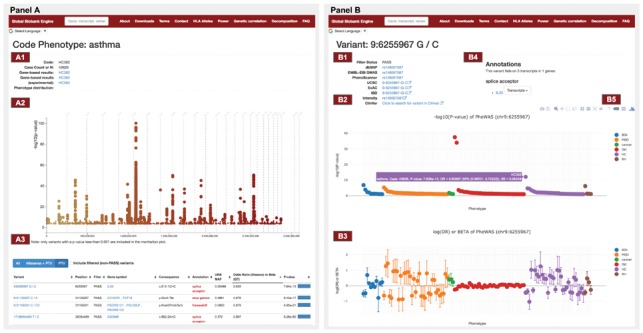
Screenshots of phenotype page (left) and variant page (right). Shown here is the phenotype page for asthma in the UK Biobank and the variant page for the protein-truncating variant rs146597587 in *IL33* found to protect against asthma. (A1) Summary of phenotype information including sample count and links to other analyses. (A2) Manhattan plot displaying significance of association of each variant. (A3) Detailed variant information is summarized in a table. (B1) Variant summary and link-outs to external references. (B2) Manhattan plot for a PheWAS. Phenotypes are binned by category. (B3) Effect size estimate plot of the log (OR) for each phenotype. (B4) Variant annotations and links to associated genes. (B5) Figures can manipulated using the tools provided

The phenotype page presents a summary of the results of a GWAS run for a phenotype of interest. The first part of the page displays relevant data such as the sample count included in the GWAS as well as links to other analyses related to this phenotype (Fig. 1.A1). Next, the Manhattan plot is displayed including all variants with *P*-value < 0.001 (Fig. 1.A2). Finally, a table is included with detailed information for each variant is included. The table can be subsetted by all variants, protein truncating variants (PTVs) only, or both PTVs and missense variants.

### 2.2 Variant page

The variant page presents the annotation of a genetic variant (Fig. 1.B4), links to external resources (Fig. 1.B1) and two plots summarizing the results from PheWAS analysis of the variant. The PheWAS Manhattan plot on the top presents the statistical significance of associations (Fig. 1.B2) while the effect size plot on the bottom presents the log odds-ratio and regression coefficient for binary and continuous traits, respectively (Fig. 1.B3). The phenotypes in the plots are sorted by their category and can be subset by *P*-values. One can export the plots to image files to facilitate scientific communication (Fig. 1.B5).

### 2.3 Gene page

The gene page presents a summary of all genotype–phenotype statistics related to a single gene. This page includes a Manhattan plot which displays each variant in the gene region and the phenotype with the lowest *P*-value for that variant as well as a table summarizing additional variant information. The page also includes a figure showing the top five most related phenotypes by a rare variant aggregate analysis, MRP (DeBoever *et al.*, 2018b). The MRP results are generated using coding variants with less than 1% minor allele frequency for each gene.

### 2.4 Genetic correlation page

GBE includes an interactive application for browsing genetic correlation estimates for pairs of traits from the UK Biobank. Genetic correlations have been estimated by applying the multi-variate polygenic mixture model (MVPMM) to GWAS summary statistics for more than one million pairs of traits and can be visualized using the app (DeBoever *et al.*, 2018b). Users can select phenotypes of interest and filter results that are displayed by the app by applying statistical thresholds. MVPMM also estimates other genetic parameters including polygenicity and scale of effects which can be seen by mousing over the plot.

### 2.5 HLA alleles page

The HLA alleles page shows posterior probabilities of causal associations between 175 HLA allelotypes and 270 diseases in the UK Biobank. For each allelotype there is a plot showing the log odds ratio with a 95% confidence interval for each associated phenotype with posterior probability greater than 0.7. Users can also view donut charts displaying the frequencies of allelotypes at each locus.

For more detailed description of all the analyses available please see the website FAQ (https://biobankengine.stanford.edu/faq).

## 3 Implementation

GBE extends the ExAC browser (Karczewski *et al.*, 2017) which is built in Python, utilizes Flask framework and uses d3 and plot.ly for plot rendering. One change made in our implementation is the use of a SciDB backend to host the summary statistic data presented in the browser (Rivers, 2017). We found SciDB to have superior performance with the large amount of data that needs to be stored and queried.

## 4 Availability

GBE browsing capabilities are now publicly available at biobankengine.stanford.edu.

## 5 Future directions

GBE is under active development. Here, we describe several areas of improvement. We are developing improved search functionality for phenotypes and variants; current search is limited by availability of variants and phenotypes within the database. We aim to incorporate more genetic annotations and filtering options, such as filtering by regulatory regions. At this time the data hosted within GBE is limited to the UK Biobank, we are working to streamline the incorporation of more data sources. As more biobanks come online we aim to include summary statistics from any available source. Finally, we plan to open source the GBE code repository in order to allow users to create their own private version of GBE.
